# Small world of the miRNA science drives its publication dynamics

**DOI:** 10.18699/VJGB-22-100

**Published:** 2022-12

**Authors:** A.B. Firsov, I.I. Titov

**Affiliations:** A.P. Ershov Institute of Informatics Systems of the Siberian Branch of the Russian Academy of Sciences, Novosibirsk, Russia; Institute of Cytology and Genetics of the Siberian Branch of the Russian Academy of Sciences, Novosibirsk, Russia Novosibirsk State University, Novosibirsk, Russia

**Keywords:** k-mer, n-gram, miRNA, digital library, organization co-authorship, small world, k-mer, n-gram, миРНК, электронная библиотека, соавторство организаций, малый мир

## Abstract

Many scientific articles became available in the digital form which allows for querying articles data, and specifically the automated metadata gathering, which includes the affiliation data. This in turn can be used in the quantitative characterization of the scientific field, such as organizations identification, and analysis of the co-authorship graph of those organizations to extract the underlying structure of science. In our work, we focus on the miRNA science field, building the organization co-authorship network to provide the higher-level analysis of scientific community evolution rather than analyzing author-level characteristics. To tackle the problem of the institution name writing variability, we proposed the k-mer/n-gram boolean feature vector sorting algorithm, KOFER in short. This approach utilizes the fact that the contents of the affiliation are rather consistent for the same organization, and to account for writing errors and other organization name variations within the affiliation metadata field, it converts the organization mention within the affiliation to the K-Mer (n-gram) Boolean presence vector. Those vectors for all affiliations in the dataset are further lexicographically sorted, forming groups of organization mentions. With that approach, we clustered the miRNA field affiliation dataset and extracted unique organization names, which allowed us to build the co-authorship graph on the organization level. Using this graph, we show that the growth of the miRNA field is governed by the small-world architecture of the scientific institution network and experiences power-law growth with exponent 2.64 ± 0.23 for organization number, in accordance with network diameter, proposing the growth model for emerging scientific fields. The first miRNA publication rate of an organization interacting with already publishing organization is estimated as 0.184 ± 0.002 year–1.

## Abstract (ru)

Многие научные статьи стали доступны в цифровом виде, что позволяет запрашивать данные статей и, в частности, автоматически собирать метаданные, включая данные об аффилиации. Это, в свою очередь, можно использовать для количественных оценок научной области, например для идентификации организаций и анализа графа соавторства этих организаций для извлечения базовой структуры науки. В настоящей работе рассмотрена область исследования микроРНК, а именно граф соавторства организаций и анализ его эволюции. Чтобы решить проблему вариативности написания названия организаций, был предложен алгоритм сортировки логических векторов признаков k-mer/n-gram. В нем используется тот факт, что содержание аффилиации довольно консистентно для одной и той же организации. Для учета ошибок написания и других артефактов названия организации в поле метаданных аффилиации наш подход преобразует упоминание организации внутри аффилиации в K-Mer (n-gram) булевый вектор присутствия. Далее векторы всех аффилиаций из набора данных лексикографически сортируются, образуя группы упомина- ний организаций. Таким подходом был кластеризован набор данных аффилиаций в области исследования микроРНК и определены названия уникальных организаций, что позволило построить граф соавторства на уровне научных организаций. С помощью этого графа показано, что рост области исследования микроРНК контролируется архитектурой малого мира сети научных организаций и испытывает степенной рост с по- казателем степени 2.64 ± 0.23 для числа организаций в соответствии с диаметром сети, предлагая модель роста новых научных направлений. Скорость публикации первой статьи по микроРНК у организации при ее взаимодействии с другой организацией, уже публиковавшейся в этой области, аппроксимируется как 0.184 ± 0.002 год–1.

## Introduction

Scientific structures stimulate the productivity of scientific work by providing researchers with material and technical conditions and a scientific environment. One of the factors for the effectiveness of scientific work is the interaction of researchers in the form of an exchange of ideas or joint work and is manifested in the form of scientific publications coauthorship. Analysis of the co-authorship of research institutions, rather than characteristics at the authors level, makes it possible to provide a higher-level analysis of the evolution of the scientific community, in particular the organization of “invisible colleges” or the development of international cooperation on a global scale (Leydesdorff et al., 2013). Such studies are aimed at finding the reasons for competition and cooperation in specific areas of research (Wagner, Leydesdorff, 2005), as well as identifying patterns of international publication activity (Ribeiro et al., 2017). In general, in order to understand the structure of the scientific community and the process of knowledge spreading in the field of science, analysis should be carried out both at the author level and at the organization level.

A graph is a small world if L log(N ), where L is the average shortest distance of the graph, N is the number of graph vertices. In other words, any two vertices are reachable from the other through a small number of hops through other vertices, but the probability that they are adjacent is small.

This type of networks are found in many real-world phenomena, such as the spread of the infection (Liu et al., 2015), neural connections (Muldoon et al., 2016), etc. The analysis of the effect of the small world in the knowledge spreading (Shi, Guan, 2016) is of particular interest, and therefore our study aims to check whether the interaction graph of organizations in the miRNA research field is a small world

Since in a small world the vertices are reachable between each other via a small number of hops, processes such as the spread of the infection or knowledge must occur differently than in a regular graph.

To determine that a graph is a small world, various criteria have been proposed in several works (Watts, Strogatz, 1998; Newman et al., 2000). In our work, we chose a categorical criterion to identify the small world effect in a network of microRNA organizations co-authorship, following (Humphries, Gurney, 2008), where the authors introduced a measure of the “small-world-ness”:

**Formula. 1.**
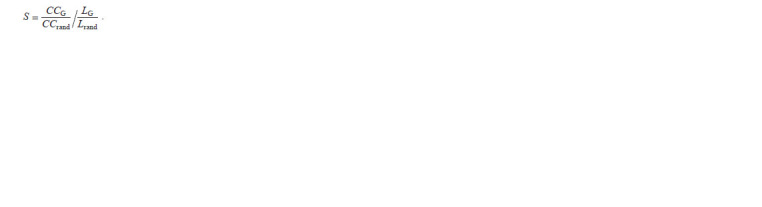
Formula. 1.

In the equation above, CCG is the clustering coefficient of graph G, LG is the average length of the shortest paths of graph G, CCrand and Lrand are the parameters of a random graph with random uniform edge placement with the same number of nodes and edges as graph G.

The knowledge spreading process can be interpreted as a process of “information contagion” where, through an intermediate host (scientific publications), organizations can be inspired by a particular area of research and start publishing articles themselves. Such a process can be modeled using the Susceptible, Infectious, Recovered (SIR) model (Goffman, Newill, 1964). Within the framework of this model, a system of differential equations is compiled that simulates the dynamics of infection and recovery of subjects. In the simplest case of a homogeneous environment, the solution to these equations at short times is the exponential growth in the number of infected subjects.

In (Vazquez, 2006), the author models the incidence rate using the SIR model for problems where transmission graphs are known and have the small world property (Muldoon et al., 2016). The author adapts the SIR propagation model to a spanning tree (AST) representation of the original graph and obtains the exact normalized incidence rate for the AST, ρ(t), which approximates this rate for the original graph. Thus, given that the graph has the small world property, there is an exact solution to the normalized infection rate for the AST, which is the approximation for the original graph:

**Formula. 2.**
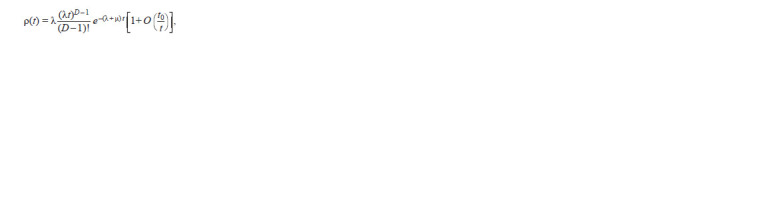
Formula. 2.

where λ and μ are, respectively, the rates of infection and recovery within the framework of the SIR model, D is the average shortest distance of the graph, t0 is the transition time between modes. The graph, in addition to having the characteristics of a small world, must satisfy one of the conditions for γ (the exponent of the power law distribution of degrees of vertices) and υ (the Pearson correlation coefficient of the degree between pairs of connected nodes) (Vazquez, 2006):

**Formula. 3.**
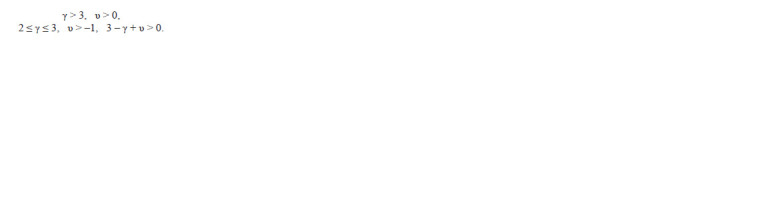
Formula. 3.

## Materials and methods

The PubMed digital library was used to collect the miRNA reseacrh area affiliation dataset. From these affiliations, mentions of the organizations were extracted. To do this, a keyword-based approach was used to identify which part of the affiliation contains what information about the mention of the organization (organization name, country, city, etc.).

An example of splitting an affiliation into mentions of organizations with a country identification for an article with PubMed ID 19996210 (1) Authors’ Affiliations: Cancer Genetics, Kolling Institute of Medical Research; Department of Endocrinology; Department of Anatomical Pathology, Royal North Shore Hospital, St. Leonards, New South Wales, Australia; Department of Surgery, Bankstown Hospital, Bankstown, New South Wales, Australia; South Western Sydney Clinical School, University of New South Wales; Endocrine Surgical Unit, University of Sydney; Department of Surgery, Liverpool Hospital, Sydney, New South Wales, Australia; Endocrine Surgical Unit, University of California Los Angeles; and Division of Hematology and Oncology, Department of Medicine, University of California Los Angeles School of Medicine, Los Angeles, California. 1. kolling institute of medical research, Australia 2. royal north shore hospital, Australia 3. bankstown hospital, Australia 4. university of new south wales, Australia 5. university of sydney, Australia 6. liverpool hospital, Australia 7. university of california los angeles, UNKNOWN 8. university of california los angeles, school of medicine, UNKNOWN

Then, for all these mentions, a dictionary of unique K-Mers (n-grams) was built, where K = 2, and for each mention, a Boolean vector of the presence of a certain K-Mer in this mention was formed. Next, these mention vectors were sorted lexicographically to obtain a list of vectors, in which similar mentions are grouped by design. After that, for each adjacent pair of mentions, the distance according to the Dice metric was calculated, and if it exceeded the specified threshold, this was the evidence that the mentions belong to different clusters, which gives us a grouping of mentions (see the Table).

**Table 1.**
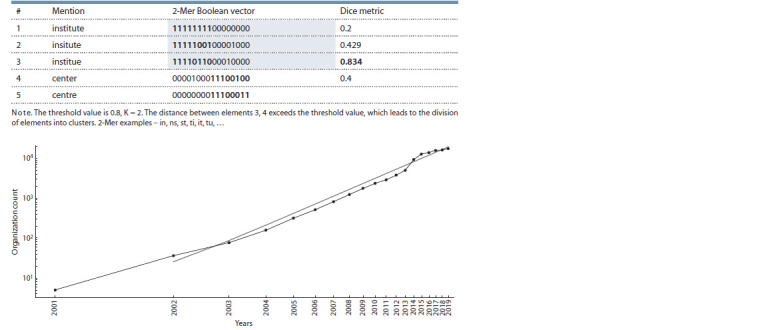
An example of organizations identification Annual number of organizations that published an article in the field of the microRNA research as a function of time in double logarithmic coordinates.

These grouped mentions contain references to the same organization; so, in the next step, we can build an organization co-authorship graph by identifying which organizations published the same article together.

## Results

The analysis of the structural characteristics of the graph of scientific organizations in the miRNA research field shows that this graph satisfies the criteria of a small world (Muldoon et al., 2016) with the exponent of the degree of power distribution γ = 2.01 and the assortativity coefficient of the degrees of graph vertices υ = –0.03. Therefore, for the number of scientific organizations with publications in the field, one can expect a power-law growth according to the model (Vazquez, 2006). The model (Vazquez, 2006) states that the initial growth in the number of vertices has a power-law dependence with the exponent D – 1, where D is the average length of the shortest paths in the graph. For the graph of scientific organizations of the microRNA research field D = 3.46, and the approximated power parameter D – 1 = 2.64 ± 0.23 (see the Figure), which gives a deviation of about 7 % from what is predicted by the model.

Approximation of the “information contagion” rate gives the rate λ = 0.184 ± 0.002 year–1, which characterizes the rate of the first microRNA publication by an organization in coauthorship with another organization that already published in this field.

Analysis of the subgraph of Russian scientific institutions in the miRNA research field shows that the activity of Russian organizations is inferior to the average activity of organizations in the field (the average number of publications per organization is 0.92 in Russia against 21.5 on average in the field). At the same time, the Russian community turns out to be denser: the clustering coefficient of the subgraph of Russian organizations exceeds the average for the field with the value of 0.708 for Russian organizations compared to the 0.361 for the microRNA field average. The US is Russia’s most active partner in international cooperation with 50 joint publications. However, US-Russian cooperation is unstable and decentralized, and the leaders in active cooperation with Russian organizations are the German Center for Cancer Research, Harbin Medical University, and Karolinska Institute (6 joint publications each).

## Discussion

Understanding the productivity factors of research organizations and the dynamics of their publication activity is important for science management. In addition to algorithms for automatic identification of organizations, projects such as ror.org are actively developing, and are aimed at identifying scientific institutions by assigning unique identifiers to them (similar to orcid.org for authors). These projects simplify the identification of organizations but require the acceptance of the use of such projects by the authors of publications, since in order to be able to fully identify each organization, it is necessary to indicate the ror.org identifier for each affiliation from the publication, which cannot currently be guaranteed. Therefore, in the near future, automatic identification algorithms for organizations will stay relevant

In our work, the data presented was gathered as of 2019, and at the current moment the structure of the graph could change. In addition, the data in the PubMed library can be updated retrospectively. Nevertheless, data from publications as of January 23, 2022 show that the picture of the evolution of the miRNA field has not fundamentally changed (data not shown). The new geopolitical reality will inevitably affect the structure of interaction and co-authorship in scientific fields. However, due to the time delay in the visible results of cooperation, a change in scientific cooperation will not appear in the databases until 2024.

## Conclusion

One of the models of the development of new knowledge areas is the “information contagion” model, in which new ideas are randomly distributed among researchers, infecting more and more of them (Goffman, Newill, 1964). The distribution law can be determined by the structure of the environment. In this work, it was shown that the organization co-authorship graph in the microRNA research field is a small world and, as a result, the publication activity of the area demonstrates a power-law growth according to the model (Vazquez, 2006). The slower than exponential growth occurs due to the “self-avoidance” of propagation paths in compact networks of the small world: when the next node of the small world is “infected” with information, there is a high probability that this node has already been “infected” by an alternative path. The co-authorship graph for our analysis was built using the organization mention clustering algorithm based on sorting K-Mer boolean feature vectors (KOFER).

## Conflict of interest

The authors declare no conflict of interest.
